# Risk of incident obstructive sleep apnoea in patients with type 1 diabetes: a population-based retrospective cohort study

**DOI:** 10.1007/s00125-022-05714-5

**Published:** 2022-05-24

**Authors:** Ziyad Alshehri, Anuradhaa Subramanian, Nicola J. Adderley, Krishna M. Gokhale, Muhammad Ali Karamat, Clare J. Ray, Prem Kumar, Krishnarajah Nirantharakumar, Abd A. Tahrani

**Affiliations:** 1 Institute of Clinical Sciences, University of Birmingham, Birmingham, UK; 2 Respiratory Therapy Department, Taibah University, Medina, Saudi Arabia; 3 Institute of Applied Health Research, University of Birmingham, Birmingham, UK; 4 Department of Diabetes and Endocrinology, University Hospitals Birmingham NHS Foundation Trust, Birmingham, UK; 5 Institute of Metabolism and Systems Research, University of Birmingham, Birmingham, UK; 6 Centre for Endocrinology, Diabetes and Metabolism, Birmingham Health Partners, Birmingham, UK

**Keywords:** Depression, Obesity, Sleep apnoea, Type 1 diabetes

## Abstract

**Aims/hypothesis:**

People with type 2 diabetes are at increased risk of developing obstructive sleep apnoea. However, it is not known whether people with type 1 diabetes are also at an increased risk of obstructive sleep apnoea. This study aimed to examine whether people with type 1 diabetes are at increased risk of incident obstructive sleep apnoea compared with a matched cohort without type 1 diabetes.

**Methods:**

We used a UK primary care database, The Health Improvement Network (THIN), to perform a retrospective cohort study between January 1995 and January 2018 comparing sleep apnoea incidence between patients with type 1 diabetes (exposed) and without type 1 diabetes (unexposed) (matched for age, sex, BMI and general practice). The outcome was incidence of obstructive sleep apnoea. Baseline covariates and characteristics were assessed at the start of the study based on the most recent value recorded prior to the index date. The Cox proportional hazards regression model was used to estimate unadjusted and adjusted hazard ratios, based on a complete-case analysis.

**Results:**

In total, 34,147 exposed and 129,500 matched unexposed patients were included. The median follow-up time was 5.43 years ((IQR 2.19–10.11), and the mean BMI was 25.82 kg/m^2^ (SD 4.33). The adjusted HR for incident obstructive sleep apnoea in patients with type 1 diabetes vs those without type 1 diabetes was 1.53 (95% CI 1.25, 1.86; *p*<0.001). Predictors of incident obstructive sleep apnoea in patients with type 1 diabetes were older age, male sex, obesity, being prescribed antihypertensive or lipid-lowering drugs, atrial fibrillation and depression.

**Conclusions/interpretation:**

Individuals with type 1 diabetes are at increased risk of obstructive sleep apnoea compared with people without diabetes. Clinicians should suspect obstructive sleep apnoea in patients with type 1 diabetes if they are old, have obesity, are male, have atrial fibrillation or depression, or if they are taking lipid-lowering or antihypertensive drugs.

**Graphical abstract:**

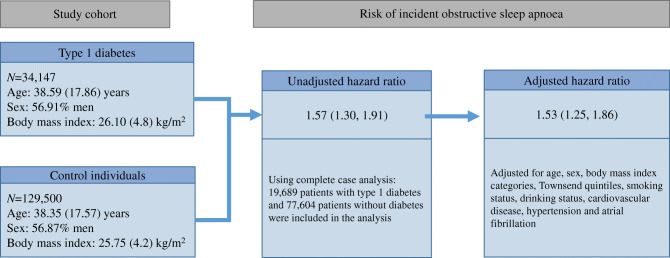

**Supplementary Information:**

The online version of this article (10.1007/s00125-022-05714-5) contains peer-reviewed but unedited supplementary material.

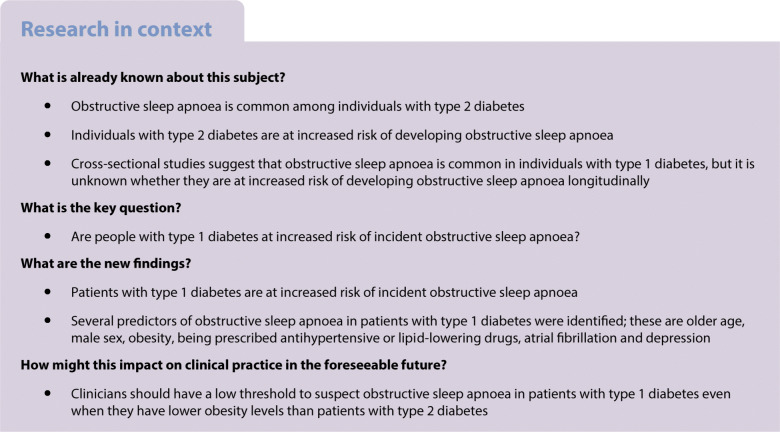



## Introduction

Obstructive sleep apnoea is common, affecting 10% of women and 20% of men in high-income countries [1]. Obstructive sleep apnoea is characterised by repeated complete upper airway obstruction (apnoea) or partial upper airway obstruction (hypopnoea), leading to recurrent episodes of oxygen desaturation and resaturation, as well as cyclical changes in heart rate, blood pressure and sympathetic activity, and disruption of sleep architecture [2, 3]. Obstructive sleep apnoea has been found to be associated with an increased risk of road traffic accidents, reduced workplace productivity, type 2 diabetes, insulin resistance, hypertension, CVD, reduced quality of life, and increased mortality risk [4–11].

Obstructive sleep apnoea is a well-established risk factor for the development of type 2 diabetes [12]. More recently, our group has shown that patients with type 2 diabetes are also at increased risk of developing obstructive sleep apnoea, suggesting that the relationship between obstructive sleep apnoea and type 2 diabetes is bi-directional [13]. We have also demonstrated previously that obstructive sleep apnoea in people with type 2 diabetes is associated with increased oxidative stress, nitrosative stress, poly-ADP ribose polymerase activation and endothelial dysfunction in cross-sectional studies [14, 15], and is associated with diabetes-related micro- and macrovascular complications in cohort studies [16–18]. However, little is known about obstructive sleep apnoea in patients with type 1 diabetes. It is important to ascertain whether people with type 1 diabetes are at increased risk of obstructive sleep apnoea given the significant above-mentioned comorbidities associated with obstructive sleep apnoea [15–17, 19, 20].

It is plausible that patients with type 1 diabetes may be at increased risk of obstructive sleep apnoea due to the increasing prevalence of obesity and insulin resistance in patients with type 1 diabetes and the high prevalence of autonomic neuropathy, which may contribute to obstructive sleep apnoea pathogenesis by affecting upper airways control [21–24]. Several small cross-sectional studies reported a high prevalence of obstructive sleep apnoea in patients with type 1 diabetes (10–46%) [25–28]. Other studies have shown a higher prevalence of sleep disruption and obstructive sleep apnoea in paediatric patients with type 1 diabetes compared with controls, that experimental sleep restriction reduced insulin sensitivity in type 1 diabetes, and that sleep apnoea and sleep disruption were associated with worse glycaemic control [29–33]. However, the direction of these associations is not clear, and hence longitudinal studies are needed.

Therefore, we performed a cohort study with the primary aim of assessing whether patients with type 1 diabetes are at increased risk of developing incident obstructive sleep apnoea compared with matched controls without diabetes. A secondary aim was to identify predictors of incident obstructive sleep apnoea in the patients with type 1 diabetes.

## Methods

### Study design

We used a UK primary care database, The Health Improvement Network (THIN), to perform a retrospective cohort study between January 1995 and January 2018 comparing sleep apnoea incidence between patients with type 1 diabetes (exposed) and without type 1 diabetes (unexposed) (matched for age, sex, BMI and general practice).

### Data source

The datasets for this study were extracted from The Health Improvement Network (THIN) database, which is a nationally representative electronic primary care database of more than 15 million patients from 787 practices in the UK [18]. This database contains anonymised medical records and is generalisable to the UK population. The data include coded demographic details, symptoms and diagnoses, all prescribed drugs, and the results of diagnostic tests [34, 35]. The database has previously been used in multiple studies of type 1 diabetes or sleep apnoea [13, 36].

### Study population

To ensure that the data extracted were of good quality, we included patients from practices after at least one year of acceptable mortality reporting. Acceptable mortality reporting is important for epidemiological studies as under-reporting may result in attempted follow-up of participants who are actually dead. In contrast, over-reporting of mortality may result in reporting deaths in a specific period for people who actually died before the reporting dates [37]. Patients were only included from these practices if they have been registered with that practice for at least 1 year, to ensure completeness of the data.

In this study, we compared the HR of incident obstructive sleep apnoea in all age groups in patients with type 1 diabetes (exposed) vs matched patients without a diagnosis of type 1 diabetes (controls/unexposed).

Type 1 diabetes diagnosis was ascertained by the presence of any clinical (Read) code consistent with type 1 diabetes (https://digital.nhs.uk/article/1104/Read-Codes) in the absence of any record of type 2 diabetes or type 2 diabetes medications (except metformin) throughout the study period. The control (unexposed) cohort had no codes relating to any type of diabetes throughout the study period. Each exposed patient was matched to a maximum of four controls by age ± 3 years, sex, BMI ± 3 kg/m^2^ and general practitioner. Exposed and control patients were eligible for inclusion in the study if they had no diagnosis of obstructive sleep apnoea at or prior to the index date.

The index date for the exposed group was the later of the type 1 diabetes diagnosis date or 12 months after joining the general practitioner. The controls were assigned the same index date that was given to their corresponding exposed patient with type 1 diabetes.

The outcome measure (obstructive sleep apnoea) was also defined based on clinical (Read) codes (see electronic supplementary material [ESM] Table 1; https://digital.nhs.uk/article/1104/Read-Codes). The cohort was followed-up until the earliest of the following: developing obstructive sleep apnoea, death, leaving the practice, the practice ceasing to contribute to this primary care database, or the study end date.

### Ethics

Data were obtained from IQVIA Medical Research Data UK, incorporating data from The Health Improvement Network, which is a registered trademark of Cegedim in the UK and other countries. Reference made to The Health Improvement Network database is intended to be descriptive of the data asset licensed by IQVIA. This work uses de-identified data provided by patients as a part of their routine primary care. This study was approved by the Scientific Review Committee (SRC) on 3rd December 2019 (SRC reference number 19THIN085).

### Statistical analysis

The baseline characteristics were summarised for those exposed and unexposed using appropriate descriptive statistics. Baseline covariates and characteristics were assessed at the start of the study. Baseline measures were the most recent value recorded prior to the index date. Continuous variables were reported as means (SD), and categorical variables were reported as the number of individuals (percentage). Crude HR and adjusted HR and their corresponding 95% CIs for incident obstructive sleep apnoea in the exposed vs unexposed groups were calculated using Cox proportional hazards regression. The Cox regression model was adjusted for several covariates including age, sex, BMI category, Townsend deprivation quintile, smoking status, drinking status, CVD, hypertension and atrial fibrillation. Bioplausibility and previous literature were used to identify covariates that were included in the multivariable model [13]. We performed a post hoc Cox regression analysis by including variables from the type 1 diabetes predictor analysis (adding atrial fibrillation, antihypertensive and lipid-lowering drugs to the Cox model). The proportional hazards assumption was tested using a log−log plot and Schoenfeld residuals test. Due to the presence of missing data for key covariates (BMI categories, Townsend deprivation quintile, smoking status, drinking status), we performed the analysis using complete-case analysis. We also performed a sensitivity analysis using multiple imputation analysis to adjust for the bias of missing data, and the results of the imputed analysis are presented in ESM Table 2.

Several sensitivity analyses were performed by limiting the analysis to patients younger than 60 years old; those younger than 60 years old and diagnosed with diabetes before the age of 40 years; and patients less than 40 years old. In addition, a secondary analysis limited to adult patients (age ≥ 18 years old) was performed. Additional subgroup analysis compared the incidence of obstructive sleep apnoea among patients with or without type 1 diabetes stratified by age, sex, BMI, Townsend deprivation quintile and comorbid conditions (CVD, hypertension, atrial fibrillation, anxiety and depression). All analyses included the exposed cohort with their corresponding controls.

Further analysis (using multiple imputation) limited to exposed participants with type 1 diabetes was performed to identify obstructive sleep apnoea risk factors (predictors) in patients with type 1 diabetes. The predictors were based on baseline variables.

All analyses were performed in Stata IC version 15 (StataCorp, USA). Two-sided *p* values were obtained, and *p* value <0.05 was considered statistically significant.

## Results

### Baseline characteristics

We identified 34,147 patients with type 1 diabetes without a diagnosis of obstructive sleep apnoea who were eligible for inclusion in the study. These were matched to 129,500 patients without diabetes or obstructive sleep apnoea. The baseline characteristics are summarised in Table 1. The study population was young, with slightly more men than women, and a low prevalence of obesity. The study population were mostly white people of European extraction, with poor glycaemic control and a relatively low number with prescriptions of CVD medications. All these features are consistent with what is expected in a type 1 diabetes population.

**Table 1:** Baseline characteristics for patients with type 1 diabetes (exposed) and patients without type 1 diabetes (controls)

	Controls	Exposed
Characteristic	(*n* = 129,500)	(*n* = 34,147)
Age (years)	38.35 (17.57)	38.59 (17.86)
Male	73,648 (56.87%)	19,434 (56.91%)
BMI (kg/m^2^)	25.75 (4.2)	26.10 (4.8)
BMI categories		
Underweight (<18.5 kg/m^2^)	799 (0.62%)	357 (1.05%)
Normal weight (18.5–25 kg/m^2^)	47,743 (36.87%)	12,079 (35.37%)
Overweight (25–30 kg/m^2^)	38,241 (29.53%)	9715 (28.45%)
Obese (>30 kg/m^2^)	15,584 (12.03%)	4919 (14.41%)
No data	27,133 (20.95%)	7077 (20.73%)
Smokers		
Non-smoker	61,798 (47.72%)	16,851 (49.35%)
Discontinued smoking	16,902 (13.05%)	5033 (14.74%)
Smoker	28,678 (22.15%)	7641 (22.38%)
No data	22,122 (17.08%)	4622 (13.54%)
Alcohol intake^a^		
Non-drinker	15,548 (12.01%)	5754 (16.85%)
Drinker	73,861 (57.04%)	17,928 (52.50%)
Excessive drinker	4539 (3.51%)	1532 (4.49%)
No data	35,552 (27.45%)	8933 (26.16%)
Townsend		
1st (least deprived)	27,400 (21.16%)	6372 (18.66%)
2nd quintile	24,102 (18.61%)	6042 (17.69%)
3rd quintile	24,027 (18.55%)	6387 (18.70%)
4th quintile	21,525 (16.62%)	5929 (17.36%)
5th (most deprived)	14,832 (11.45%)	4313 (12.63%)
No data	17,614 (13.60%)	5104 (14.95%)
Ethnicity^b^		
White	45,528 (35.16%)	15,750 (46.12%)
Black	1267 (0.98%)	362 (1.06%)
South Asians	1851 (1.43%)	365 (1.07%)
Others	421 (0.33%)	139 (0.41%)
Mixed race	808 (0.62%)	130 (0.38%)
No data	79,625 (61.49%)	17,401 (50.96%)
eGFR (ml min^−1^ [1.73 m]^−2^)		
>90 (stage 1)	19,767 (15.26%)	13,769 (40.32%)
60–90 (stage 2)	15,401 (11.89%)	7409 (21.70%)
30–59 (stage 3)	2220 (1.71%)	1849 (5.41%)
<30 (stage 4)	138 (0.11%)	436 (1.28%)
No data	91,974 (71.02%)	10,684 (31.29%)
Medication		
ACE inhibitor	6984 (5.39%)	8340 (24.42%)
Lipid-lowering drugs	6329 (4.89%)	8666 (25.38%)
Antihypertensive drugs	21,405 (16.53%)	10,774 (31.55%)
CVD	4483 (3.46%)	2629 (7.70%)
Heart failure	569 (0.44%)	459 (1.34%)
IHD	3065 (2.37%)	1798 (5.27%)
Stroke and TIA	1463 (1.13%)	950 (2.78%)
Hypertension	10,252 (7.92%)	5761 (16.87%)
Atrial fibrillation	950 (0.73%)	300 (0.88%)
Mental health		
Anxiety	13,005 (10.04%)	3172 (9.29%)
Depression	19,388 (14.97%)	6199 (18.15%)
Serious mental illness	1499 (1.16%)	447 (1.31%)
Diabetes-related variables		
HbA_1c_		
≤47.5 mmol/mol (≤ 6.5%)		1911 (5.60%)
47.5–58.5 mmol/mol (6.5–7.5%)		4664 (13.66%)
58.5–69.4 mmol/mol (7.5–8.5%)		5755 (16.85%)
≥69.4 mmol/mol (≥ 8.5%)		12,331 (36.11%)
No data		9486 (27.78%)
Diabetes treatment		
Insulin		34,147 (100%)
Metformin		2181 (6.39%)
Other diabetes drugs^c^		0 (0.00%)
Hypoglycaemia		5422 (15.88%)
Foot disease^d^		4104 (12.02%)
Retinopathy^e^		
No retinopathy		29,725 (87.05%)
R2, R3, M1		2239 (6.56%)
Blindness, laser treatment or vitreous injection		2183 (6.39%)

Data are means (SD) or number of cases (%)

^a^Excessive alcohol intake was based on Read codes that reflect heavy drinking or alcohol abuse and misuse

^b^Black includes African and Caribbean. Others include Chinese and Middle-Eastern

^c^Other diabetes drugs include acarbose, dipeptidyl peptidase-4 inhibitors, thiazolidinediones, glinides, glucagon-like peptide-1 analogues, sulfonylureas and sodium-glucose cotransporter-2 inhibitors

^d^Foot disease includes limb amputation, foot ulcer, gangrene, peripheral neuropathy and peripheral vascular disease

^e^Retinopathy is defined according to the NSC-UK grading: R2 is pre-proliferative, R3 is proliferative, M1 is maculopathy [49]

Townsend, material deprivation score; IHD, ischaemic heart disease; TIA, transient ischaemic attack

### Type 1 diabetes and incident obstructive sleep apnoea

By the end of the study, obstructive sleep apnoea had been diagnosed in 219 (0.64%) of the patients with type 1 diabetes, and 531 (0.41%) of the controls, over a median follow-up duration of 5.43 years (IQR 2.19–10.11). The median diabetes duration in patients with type 1 diabetes who were diagnosed with obstructive sleep apnoea was 19.36 years (IQR 7.42–28.11), which was approximately double the duration for patients with type 1 diabetes without obstructive sleep apnoea (median 10.47, IQR 2.56–21.17).

A total of 19,689 patients with type 1 diabetes without a diagnosis of obstructive sleep apnoea and 77,604 patients without diabetes or obstructive sleep apnoea were included in the complete-case analysis. The incidence rates were 11.62 and 7.46 per 10,000 person-years for the exposed participants and controls, respectively. Evaluation of the incidence rate for obstructive sleep apnoea over time showed a consistent higher annual rate in patients with type 1 diabetes since 2002 (ESM Fig. 1). After adjusting for baseline age, sex, BMI category, Townsend quintile, smoking status and drinking status, the adjusted HR for obstructive sleep apnoea in the exposed vs control group was 1.51 (95% CI 1.24, 1.83; *p*< 0.001) (Table 2). Further adjustments for CVD, hypertension and atrial fibrillation at baseline did not change the findings (adjusted HR 1.53; 95% CI 1.25, 1.86; *p*<0.001) (Table 2). This association remained significant after multiple imputation of missing data (HR 1.68; 95% CI 1.43, 1.97; *p*< 0.001) (ESM Table 2). The adjusted HR decreased to 1.24 (95% CI 1.00, 1.54; *p*=0.05) after performing a post hoc analysis by adding baseline depression and use of lipid-lowering and antihypertensive drugs to the above-mentioned baseline covariates in the complete-case analysis model. The Cox proportional hazards assumption was examined using a log–log plot and Schoenfeld residuals test, and there were no violations.

**Table 2:** Cox proportional hazards models for HR of incident obstructive sleep apnoea (complete-case analysis)

	Primary analysis		Sensitivity model A		Sensitivity model B		Sensitivity model C	
Variable	Exposed	Unexposed	Exposed	Unexposed	Exposed	Unexposed	Exposed	Unexposed
Population	19,689	77,604	16,956	66,705	15,047	59,160	10,193	39,100
Number of OSA cases	139	398	121	350	104	291	40	150
Person-years	119,676	533,463	104,433	453,262	92,348	394,997	57,365	240,703
Incidence rate of OSA per 10,000 person-years	11.62	7.46	11.59	7.72	11.26	7.37	6.97	6.23
Follow-up (years), median (IQR)	4.79 (1.84–9.49)	5.82 (2.46–10.37)	4.83 (1.84–9.57)	5.67 (2.36–10.29)	4.75 (1.81–9.56)	5.50 (2.25–10.12)	3.92 (1.50–8.98)	4.75 (1.88–9.50)
Crude HR (95% CI)	1.57 (1.30, 1.91)**		1.51 (1.23, 1.86)**		1.54 (1.23, 1.93)**		1.13 (0.80, 1.60)	
Adjusted HR^a^ (95% CI)	1.51 (1.24, 1.83)**		1.45 (1.18, 1.78)**		1.47 (1.18, 1.85)**		1.09 (0.77, 1.54)	
Adjusted HR^b^ (95% CI)	1.53 (1.25, 1.86)**		1.41 (1.14, 1.74)**		1.41 (1.12, 1.78)**		0.95 (0.66, 1.37)	
Adjusted HR^c^ (95% CI)	1.24 (1.00, 1.54)*		1.17 (0.93, 1.49)		1.21 (0.94, 1.57)		0.84 (0.57, 1.25)	

Model A limited analysis to people who were <60 years old at index date along with their matched controls

Model B limited analysis to the population who were <60 years old at index date, and people diagnosed with type 1 diabetes before the age of 40 years along with their matched controls

Model C limited analysis to people who were <40 years old at the start along with their matched controls

^a^Model adjusted for age, sex, BMI category, Townsend quintile, smoking status and drinking status

^b^Model adjusted for age, sex, BMI category, Townsend quintile, smoking status, drinking status, CVD, hypertension and atrial fibrillation

^c^Post hoc analysis adjusting for age, sex, BMI category, Townsend quintile, smoking status, drinking status, CVD, hypertension, atrial fibrillation, depression, lipid-lowering drugs and antihypertensive drugs

OSA, obstructive sleep apnoea

**p<0.01; *p<0.05

### Sensitivity analysis

Limiting the analysis to people younger than 60 years old or patients younger than 60 years old who were diagnosed with type 1 diabetes before the age of 40 years, along with their respective controls, did not change the results of the main analysis (Table 2). Limiting the analysis to patients younger than 40 years of age attenuated the association between type 1 diabetes and obstructive sleep apnoea (Table 2). This attenuation is not surprising given that older age is a risk factor for obstructive sleep apnoea.

We performed an additional complete-case analysis limited to adult people (age ≥ 18 years old) with type 1 diabetes (*n*= 19,493) and their matched controls (*n*=77,018). The results for the association between type 1 diabetes and obstructive sleep apnoea for the adults were similar to the results for the whole population (ESM Table 3).

**Fig. 1:**
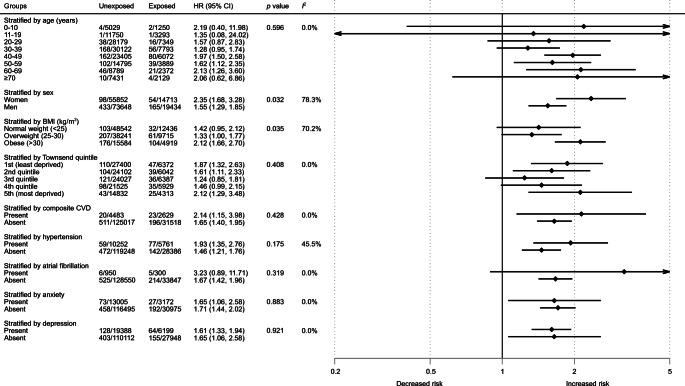
Forest plot showing adjusted HR for obstructive sleep apnoea in patients with type 1 diabetes compared with patients without diabetes in subgroups stratified by age, sex, BMI, Townsend score and comorbidity. The HR was adjusted for age, sex, BMI categories, Townsend quintiles, smoking status and alcohol intake

The stratified analysis (Fig. 1) found that patients with type 1 diabetes were more likely to develop obstructive sleep apnoea compared with people without diabetes across age categories, in men and women, in people with normal weight, overweight or obesity, across social deprivation quantiles, and in the presence or absence of comorbidities.

### Predictors of incident obstructive sleep apnoea in patients with type 1 diabetes

Male sex, age, being overweight or obese, use of lipid-lowering medication, use of antihypertensive medication, and a history of atrial fibrillation and depression were associated with increased HR of incident obstructive sleep apnoea (Table 3).

**Table 3:** Predictors of incident OSA in patients with type 1 diabetes (multiple imputation analysis)

Predictors	HR (95% CI)	*p* value
Age at diagnosis	0.97 (0.92–1.02)	0.192
Diabetes duration	0.98 (0.94–1.03)	0.531
Age groups (years)		
20–30	Reference	
0–10	0.46 (0.08–2.62)	0.381
10–20	0.12 (0.01–0.95)	0.044
30–40	2.01 (0.98–4.11)	0.055
40–50	3.06 (1.08–8.66)	0.036
50–60	2.68 (0.62–11.58)	0.186
60–70	3.21 (0.48–21.63)	0.231
70–maximum	1.95 (0.15–24.76)	0.607
Sex		
Female	Reference	
Male	2.69 (1.95–3.71)	<0.001
BMI category (kg/m^2^)		
Normal weight (<25)	Reference	
Overweight (25–30)	1.98 (1.95–3.06)	0.002
Obese (>30)	6.35 (4.17–9.65)	<0.001
Townsend		
1st quintile	Reference	
2nd quintile	0.88 (0.58–1.36)	0.572
3rd quintile	0.84 (0.53–1.31)	0.439
4th quintile	0.88 (0.56–1.39)	0.595
5th quintile	0.97 (0.59–1.59)	0.893
Alcohol intake^a^		
Non-drinker	Reference	
Drinker	1.09 (0.75–1.59)	0.640
Excessive drinker	0.83 (0.39–1.77)	0.634
Smokers		
Non-smoker	Reference	
Discontinued smoking	1.00 (0.69–1.44)	0.986
Smoker	0.96 (0.66–1.38)	0.808
HbA_1c_ category		
≤47.5 mmol/mol (≤ 6.5%)	Reference	
47.5–58.5 mmol/mol (6.5–7.5%)	1.15 (0.52–2.54)	0.728
58.5–69.4 mmol/mol (7.5–8.5%)	1.36 (0.63–2.94)	0.427
≥69.4 mmol/mol (≥8.5%)	1.54 (0.75–3.20)	0.242
No data	1.46 (0.68–3.12)	0.327
eGFR (ml min^−1^ [1.73 m]^−2^)		
>90 (stage 1)	Reference	
60–90 (stage 2)	1.12 (0.80–1.57)	0.500
30–59 (stage 3)	0.99 (0.53–1.85)	0.983
<30 (stage 4)	1.40 (0.54–3.68)	0.491
No data	0.87 (0.58–1.32)	0.520
ACR category		
<3 (mg/mmol)	Reference	
3–30 (mg/mmol)	0.69 (0.31–1.55)	0.370
>30 (mg/mmol)	1.02 (0.31–3.38)	0.977
No data	1.02 (0.70–1.48)	0.920
Hypoglycaemia	0.83 (0.58–1.20)	0.334
Foot disease^b^	1.05 (0.72–1.51)	0.813
Retinopathy^c^		
No retinopathy	Reference	
R2, R3, M1	1.04 (0.66–1.66)	0.855
Blind, laser treatment or vitreous injection	1.09 (0.70–1.71)	0.692
Medication		
Lipid-lowering drugs	1.95 (1.40–2.71)	<0.001
Antihypertensive drugs	1.46 (1.01–2.10)	0.042
Heart failure	1.59 (0.53–4.72)	0.408
IHD	0.70 (0.39–1.24)	0.220
Stroke and TIA	0.73 (0.31–1.68)	0.454
Hypertension	0.98 (0.69–1.40)	0.927
Atrial fibrillation	3.06 (1.19–7.89)	0.021
Depression	1.94 (1.38–2.73)	<0.001
Anxiety	0.95 (0.61–1.49)	0.836
Serious mental illness	1.17 (0.47–2.92)	0.743

^a^Excessive alcohol intake was based on Read codes that reflect heavy drinking or alcohol abuse and misuse

^b^Foot disease includes limb amputation, foot ulcer, gangrene, peripheral neuropathy and peripheral vascular disease

^c^Retinopathy is defined according to the NSC-UK grading: R2 is pre-proliferative, R3 is proliferative, M1 is maculopathy [49]

Townsend, material deprivation score; ACR, albumin to creatinine ratio; IHD, ischaemic heart disease; TIA, transient ischaemic attack

## Discussion

We found that patients with type 1 diabetes are at increased risk of obstructive sleep apnoea compared with patients without diabetes. The study also identified a number of predictors of incident obstructive sleep apnoea in patients with type 1 diabetes. As far as we are aware, this is the first study to examine the relationship between obstructive sleep apnoea and type 1 diabetes longitudinally.

The adjusted HRs for incident obstructive sleep apnoea in people with vs without type 1 diabetes reported in this study are similar to those reported in patients with type 2 diabetes in another study by our team that used the current database [13]. This may be surprising considering the lower prevalence of obesity and younger age of patients with type 1 vs type 2 diabetes. This finding suggests that another mechanism may play a role in the development of obstructive sleep apnoea in people with type 1 diabetes. One potential mechanism, which was not directly measured in this study, is the presence of diabetic autonomic neuropathy; this mechanism is indirectly supported in this data by the longer duration of diabetes in patients with obstructive sleep apnoea vs no obstructive sleep apnoea, and the finding that atrial fibrillation was a predictor of incident obstructive sleep apnoea, as diabetic autonomic neuropathy is also associated with longer diabetes duration, atrial fibrillation and CVD [24].

Several previous cross-sectional studies observed that obstructive sleep apnoea is common in patients with type 1 diabetes [25–28], but the direction of this relationship was not clear. In this study, we found that patients with type 1 diabetes are at increased risk of obstructive sleep apnoea. In addition, cross-sectional studies found that obstructive sleep apnoea in type 1 diabetes was associated with diabetes-related peripheral and autonomic neuropathy, chronic kidney disease and retinopathy [26, 28]. Our group has also reported previously that, in patients with type 2 diabetes, having obstructive sleep apnoea was associated with an increased incidence of micro- and macrovascular complications [18]. In addition, patients with type 1 diabetes are at increased risk of hypoglycaemia, which is important in the context of driving as obstructive sleep apnoea is also associated with an increased risk of road traffic accidents [38]. Hence, clinicians and healthcare professionals caring for patients with type 1 diabetes should be vigilant in terms of suspecting obstructive sleep apnoea in these patients despite the low prevalence of obesity and younger age, particularly patients with factors identified in our analysis as predictive of incident obstructive sleep apnoea. Future work should examine whether patients with type 1 diabetes and obstructive sleep apnoea are at increased risk of vascular complications compared with those with type 1 diabetes only.

We found that depression was an independent predictor for obstructive sleep apnoea in patients with type 1 diabetes. This is consistent with findings from non-diabetes studies. The relationship between obstructive sleep apnoea and mental health disease was examined in a large US retrospective study including over 4 million records (not specifically with diabetes), and found that depression was common in people with obstructive sleep apnoea (22%) [39]. Systematic reviews have also established the relationship between obstructive sleep apnoea and depression [40], and found that obstructive sleep apnoea treatment using continuous positive airway pressure improved depression symptoms [41].

In patients with type 1 diabetes, we also found that baseline atrial fibrillation was a strong obstructive sleep apnoea predictor. People with type 1 diabetes had an increased risk of atrial fibrillation compared with the general population in a Swedish population-based analysis [42]. Sleep disturbance has been linked to atrial fibrillation [43], and up to 85% of people with atrial fibrillation had obstructive sleep apnoea [44–47].

The study has limitations. Although the current database includes comprehensive and reliable data, the accuracy of the study depends on the quality of the data recorded by the general practitioners in the primary care clinics. Also, the severity of obstructive sleep apnoea and use of continuous positive airway pressure were not recorded in the current database. It is plausible that clinicians are more likely to look for obstructive sleep apnoea in the exposed vs unexposed cohort due to the presence of type 1 diabetes, and this may result in detection bias. Although we used certain criteria to select type 1 diabetes patients and exclude type 2 diabetes, the criteria do not eliminate the risk of misclassification. However, the study findings were robust despite matching for key variables, and in multiple sensitivity and subgroup analyses including multiple age groups and comorbidities. In this study, we used baseline characteristics to identify obstructive sleep apnoea predictors, and therefore another potential limitation is not accounting for time-varying covariates in the analysis. A further limitation of the analysis is that ethnicity was not included, due to the large proportion of patients for whom ethnicity data was missing. Previous studies also showed similar large missingness for ethnicity data [13, 18, 48].

This study is the first longitudinal study to examine the associations between type 1 diabetes, incident sleep apnoea and sleep apnoea predictors. We used a validated well-established primary care database from the UK. The breadth of the data available allowed us to adjust for a large number of variables and perform sensitivity and subgroup analyses.

In conclusion, compared with people without diabetes, patients with type 1 diabetes are at increased risk of obstructive sleep apnoea. Obesity, male sex, age, depression, atrial fibrillation, and use of lipid-lowering and antihypertensive drugs at baseline were predictors for incident obstructive sleep apnoea in type 1 diabetes. Therefore, clinicians should suspect obstructive sleep apnoea in patients with type 1 diabetes who have the above-mentioned predictors even though people with type 1 diabetes usually have less obesity than type 2 diabetes.

## Supplementary Information

ESM 1 (PDF 720 kb)

## Data Availability

THIN data governance does not allow us to share individual patient data. Researchers may apply for individual patient data access at https://www.iqvia.com/contact.

## Abbreviations

THIN: The Health Improvement Network
