# Supplementary material for: Risk of incident obstructive sleep apnoea in patients with type 1 diabetes: a population-based retrospective cohort study
Source: Diabetologia. 2022 May 24;65(8):1353–63. doi: 10.1007/s00125-022-05714-5 (PMC9283161; doi:10.1007/s00125-022-05714-5)
Supplement: Supplementary file 1 — (PDF 720 kb) [file 125_2022_5714_MOESM1_ESM.pdf]

Electronic Supplementary Material (ESM)

ESM Table 1. Read codes used to identify patients with obstructive sleep apnoea.

Read code	Description
Fy03.00	Sleep apnoea
Fy03.11	Obstructive sleep apnoea
Fy04.11	Ondine's curse
H5B..00	Sleep apnoea
H5B0.00	Obstructive sleep apnoea
R005100	[D]Insomnia with sleep apnoea
R005300	Hypersomnia with sleep apnoea
R005311	[D]Sleep apnoea syndrome
R005312	[D]Syndrome sleep apnoea
R060400	[D]Apnoea

ESM Table 2: Cox proportional hazards models for risk of incident obstructive sleep apnoea (Multiple imputation analysis)

Parameter	Exposed	Unexposed
Population	34,147	129,500
Number of OSA cases	219	531
Person-years	211,098	870,642
incidence rate of OSA per 10,000 person years	10.37	6.1
Follow-up (years), median (IQR)	4.81 [1.80-9.59]	5.60 [2.32-10.24]
Multiply imputation analysis		
Crude HR (95% CI),	1.71 (1.46-2.00)**	
Adjusted HR[a] (95% CI),	1.68 (1.43-1.97)**	
Adjusted HR[b] (95% CI),	1.68 (1.43-1.97)**	
Adjusted HR[c] (95% CI),	1.35 (1.13-1.61)**	

[a]Model adjusted for age, sex, body mass index category, Townsend quintiles, smoking status, and drinking status.

[b]Model adjusted for age, sex, body mass index category, Townsend quintiles, smoking status, drinking status, cardiovascular disease, hypertension, and atrial fibrillation.

[c]Post-hoc analysis adjusting for age, sex, body mass index category, Townsend quintiles, smoking status, drinking status, cardiovascular disease, hypertension, atrial fibrillation, depression, lipid lowering drugs, and antihypertensive drugs

OSA, obstructive sleep apnoea

**$p < 0.01$; *$p < 0.05$

ESM Table 3. Cox proportional hazards for risk of incident OSA in adult type 1 diabetes patients (exposed aged ≥ 18) and their matched controls (complete case analysis).

Parameter	Exposed	Unexposed
Population	19,493	77,018
Number of OSA cases	139	398
Person-years	118,617	529,924
incidence rate of OSA per 10,000 person years	11.86	7.8
Follow-up (years), median(IQR)	4.80 [1.84-9.50]	5.82 [2.46-10.37]
Crude HR (95% CI),	1.57 (1.30-1.91)**	
Adjusted HR[a] (95% CI),	1.51 (1.24-1.83)**	
Adjusted HR[b] (95% CI),	1.53 (1.25-1.86)**	
Adjusted HR[c] (95% CI),	1.24 (1.00-1.54) *	

[a]Model adjusted for age, sex, body mass index category, Townsend quintiles, smoking status, and drinking status.

[b]Model adjusted for age, sex, body mass index category, Townsend quintiles, smoking status, drinking status, cardiovascular disease, hypertension, and atrial fibrillation.

[c]Post-hoc analysis adjusting for age, sex, body mass index category, Townsend quintiles, smoking status, drinking status, cardiovascular disease, hypertension, atrial fibrillation, depression, lipid lowering drugs, and antihypertensive drugs.

OSA, obstructive sleep apnoea

**$p < 0.01$; *$p < 0.05$

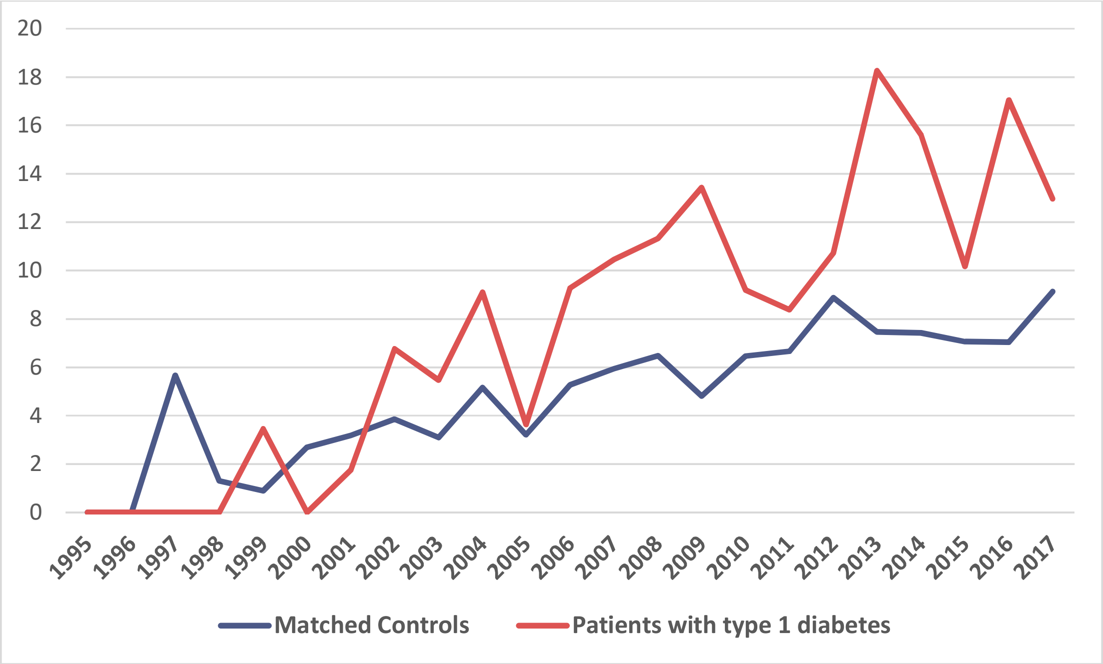

ESM Fig 1. Annual Incidence Rate of OSA Per 10,000 Person-years
